# Correction: Untangling the complexity of market competition in consumer goods – A complex Hilbert PCA analysis

**DOI:** 10.1371/journal.pone.0258600

**Published:** 2021-10-08

**Authors:** Makoto Mizuno, Hideaki Aoyama, Yoshi Fujiwara

The project title cited in the first sentence of the Acknowledgements is incorrect. The correct title is:

“Dynamics of Economy and Finance from the Economic Network Point of View” and not “Macro-Economy under COVID-19 influence: Data-intensive analysis and the road to recovery.”

The correct Acknowledgements should read: This study is conducted as a part of the Project “Dynamics of Economy and Finance from the Economic Network Point of View” undertaken at the Research Institute of Economy, Trade and Industry (RIETI). The authors are grateful for helpful comments and suggestions by Discussion Paper seminar participants at RIETI. The authors appreciate the support by INTAGE Inc. to make the data available. The authors would also like to thank participants of the conferences of Computational Social Science Japan, Japan Institute of Marketing Science, and Japan Society for Evolutionary Economics for their helpful discussions and comments. We would like to thank Editage (www.editage.com) for English language editing.
