# Supplementary material for: Untangling the complexity of market competition in consumer goods—A complex Hilbert PCA analysis
Source: PLoS One. 2021 Feb 3;16(2):e0245531. doi: 10.1371/journal.pone.0245531 (PMC7857567; doi:10.1371/journal.pone.0245531)
Supplement: S1 File — (PDF) [file pone.0245531.s001.pdf]

S1 File

Untangling the complexity of market competition in consumer goods – A complex Hilbert PCA analysis

Makoto Mizuno[1], Hideaki Aoyama[2] and Yoshi Fujiwara[3]

[1]School of Commerce, Meiji University, Tokyo 101-8301, Japan
[2] Research Institute of Economy, Trade and Industry, Tokyo 100-8901, Japan, and RIKEN iTHEMS, Wako, Saitama 351-0198, Japan
[3]Graduate School of Simulation Studies, University of Hyogo, Kobe 650-0047, Japan.

Appendix A The eigenmodes 1 and 2: detail

We list the components of the first eigenmode with absolute value above the 2σ range in Table S1 and Table S2 and, similarly, for the second eigenmode in Table S3 and Table S4.

Brand	Variable	Phase	Abs.
A1	TVAd	2.22	0.21
A1	WebV	2.57	0.07
A1	MobV	2.85	0.10
A1	Q	4.91	0.12
A1	P	5.09	0.08
A2	WebV	2.90	0.14
A2	TVAd	2.93	0.09
A2	MobV	3.08	0.08
A2	P	5.57	0.07
A3	MobV	2.59	0.18
A3	WebV	2.8	0.20
A3	TVAd	2.93	0.08
A3	Q	4.83	0.09
A3	P	5.32	0.08
A4	TVAd	0.44	0.08
A4	Q	5.29	0.07
A6	P	3.88	0.10
A6	Q	5.49	0.11
A7	P	4.85	0.11
A7	Q	5.99	0.14
B1	WebV	1.82	0.07
B1	TVAd	2.12	0.08
B1	Q	5.41	0.09
B1	P	5.83	0.07
B2	Q	3.90	0.08
B2	TVAd	4.81	0.08
B3	WebS	3.27	0.07
B3	TVAd	5.39	0.14
B4	Q	4.91	0.09

Table S1: List of components in the first eigenmode with absolute value above the 2σ range (to be continued to Table S2)

Brabd	Variable	Phase	Abs.
B5	WebV	3.52	0.08
B5	TVAd	4.18	0.15
B5	P	4.21	0.08
B5	Q	5.03	0.07
C1	WebV	3.62	0.07
C1	TVAd	4.57	0.11
C1	Q	5.33	0.08
C2	MobS	1.86	0.10
C2	MobV	1.86	0.10
C2	Q	5.63	0.11
C2	P	6.26	0.10
C3	TVAd	5.50	0.12
C3	Q	5.89	0.25
C3	P	6.16	0.27
C4	Q	0.96	0.11
C4	WebS	2.89	0.07
C4	WebV	3.37	0.09
C4	TVAd	4.68	0.11
C4	P	6.12	0.11
C5	Q	2.88	0.22
C5	P	6.01	0.33
D1	Q	5.24	0.09
D1	P	5.47	0.11
D1	TVAd	5.84	0.15
D1	WebS	6.07	0.07
D2	MobV	3.05	0.06
D2	WebV	3.54	0.07
D2	WebS	5.04	0.08
D2	P	5.52	0.09
D2	Q	5.70	0.09
D3	TVAd	3.87	0.19
D3	Q	4.77	0.07

Table S2: -continued from Table S1

Brand	Variable	Phase	Abs.
A1	TVAd	5.49	0.10
A1	Q	5.70	0.17
A1	P	5.84	0.09
A2	TVAd	4.52	0.09
A2	Q	5.73	0.11
A3	MobS	4.77	0.07
A3	TVAd	5.2	0.08
A3	Q	5.54	0.18
A3	P	5.92	0.08
A3	MobV	6.06	0.07
A4	WebS	4.09	0.08
A4	TVAd	4.76	0.13
A4	WebV	5.24	0.12
A4	Q	5.80	0.20
A4	P	6.11	0.14
A6	P	0.73	0.06
A7	Q	4.92	0.12
B1	P	5.19	0.10
B1	WebS	5.28	0.07
B1	Q	5.36	0.18
B1	TVAd	5.53	0.08
B2	Q	4.06	0.16
B2	WebS	5.02	0.07
B2	P	5.27	0.09
B2	TVAd	5.64	0.11
B3	WebV	2.91	0.08
B3	TVAd	5.21	0.09
B4	Q	5.15	0.19
B4	P	5.46	0.12

Table S3: List of components in the second eigenmode with absolute value above the 2σ range (to be continued to Table S4)

Brand	Variable	Phase	Abs.
B5	Q	5.07	0.11
B5	P	5.36	0.10
B5	TVAd	5.63	0.10
C1	WebV	2.02	0.07
C1	TVAd	5.08	0.19
C1	WebS	5.25	0.07
C1	Q	5.96	0.12
C1	P	6.28	0.08
C2	TVAd	5.22	0.12
C2	P	5.37	0.08
C2	Q	5.49	0.13
C3	Q	3.18	0.12
C3	P	3.38	0.18
C3	TVAd	3.69	0.12
C3	WebV	5.2	0.08
C4	TVAd	4.47	0.09
C4	WebV	5.14	0.07
C4	Q	5.34	0.22
C5	P	3.10	0.18
C5	Q	5.99	0.23
D1	Q	5.52	0.19
D1	WebS	5.74	0.07
D1	P	5.81	0.14
D2	WebS	5.34	0.08
D2	Q	5.34	0.18
D2	P	5.39	0.12
D2	TVAd	5.58	0.12
D3	TVAd	0.29	0.12
D3	P	5.14	0.08
D3	Q	5.81	0.21

Table S4: -continued from Table S3
